# Sclerosing Mucoepidermoid Carcinoma of the Parotid Gland

**Published:** 2016-07

**Authors:** Farahnaz Bidari-Zerehpoosh, Bijan Naghibzadeh, Elena Jamali, Moein Jamali, Amirali Mafi, Hooman Bahrami-Motlagh

**Affiliations:** 1 *Department of Pathology, Loghman Hakim Hospital, Shahid Beheshti University of Medical Sciences, Tehran, Iran.*; 2 *Department of Otorhinolaryngology, Loghman Hakim Hospital, Shahid Beheshti University of Medical Sciences, Tehran, Iran.*; 3 *Department of Chemistry, Sharif University of Technology, Tehran, Iran.*; 4 *Department of Radiology, Loghman Hakim Hospital, Shahid Beheshti University of Medical Sciences, Tehran, Iran.*

**Keywords:** Mucoepidermoid carcinoma, Sclerosing, Parotid gland

## Abstract

**Introduction:**

Mucoepidermoid carcinoma represents one of the most common malignant salivary gland tumors. However, the sclerosing morphologic variant is extremely rare with only 23 reported cases in the English-language literature since it was discovered in 1987.

**Case Report:**

Herein, we describe another case that was diagnosed in a 25-year-old woman presenting with a posterior auricular mass, as well as a review of the literature, which demonstrates that this is an extremely rare malignancy with no strict protocol for treatment.

**Conclusion:**

Pathologists must be aware of recognizing low grade sclerosing mucoepidermoid carcinoma which has metastatic potential and is frequently misdiagnosed as a benign lesion.

## Introduction

Most cases of mucoepidermoid carcinoma (MCEC) are located in the parotid gland (1). Histologically, MCEC is composed of various combinations of four cell types: mucin-producing, squamous, intermediate, and clear cells.

The better differentiated forms present grossly as a relatively well-circumscribed mass with cystic areas containing mucinous material and predominance of mucinous cells microscopically. The high-grade varieties are more solid and have a more infiltrative pattern of growth with predominance of squamoid, intermediate and clear cells over the mucin-producing cells microscopically (1). The sclerosing variant is characterized by paucity of tumor islands and intense stromal sclerosis that may obscure their typical morphological features and result in diagnostic difficulties (2). 

To date, only 24 cases of this variant have been reported in the English-language literature (3). Herein, we report another case of sclerosing mucoepidermoid carcinoma with interesting anatomical location of presentation.

## Case Report

**Fig 1:**
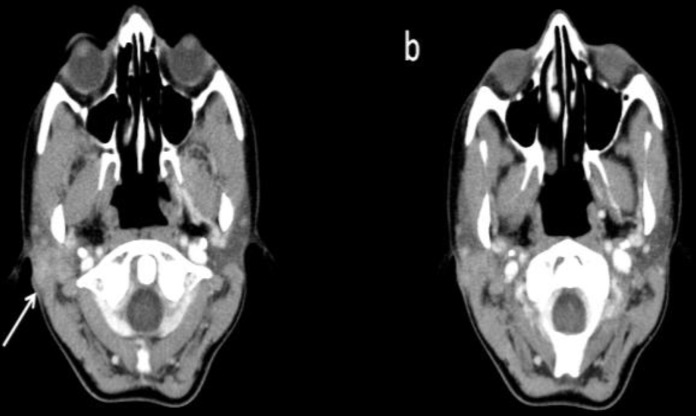
Contrast-enhanced neck CT scan: *a *is cephalad to *b* and depict a moderately enhancing mass in the posterior portion of the right parotid gland with a lobulated and partially indistinct margin. There is involvement of subcutaneous tissue (arrow

A 25-year-old woman was referred to Loghman hospital with complaint of a right posterior auricular mass since three years and a recent development of pain and increase in size. There was no palpable lump in the region of the right parotid gland, paresthesia, or evidence of cranial nerve VII dysfunction. However, an ill-defined firm mass just posterior to right earlobe at the tip of mastoid was observed. Computed tomography revealed a moderately enhanced lesion measuring 28 mm in diameter in the posterior portion of the right parotid gland (Fig.1). The decision was made to resect the mass. Intraoperatively, a firm mass was found to be mainly located at the tip of right mastoid bone with narrow connection to the parotid gland parenchyma. The gross entirety of the mass was carefully dissected from the mastoid bone to the tragus cartilage as well as parotid gland with preservation of the facial nerve.

The gross specimen, which was received in two separate formalin containers, was labeled as:

A) “parotid gland”: consisted of a portion of parotid gland tissue measuring 3*3*1.5 cm accompanied by multiple small fragments of parotid gland tissue measuring 2*2*1 cm in aggregates. The cut surface showed a relatively well-demarcated homogenous gray fibrotic mass at the periphery of the gland measuring 2 cm in greatest diameter.

B) “Posterior auricular mass”: consisted of an irregular gray elastic to fibrous tissue, measuring 1.8*1*0.6 cm, with homogenous grayish cut surfaces. 

The specimen was inked and entirely processed routinely: sections were fixed in 10% neutral-buffered formalin, processed, embedded in paraffin, sectioned, then stained with hematoxylin-eosin. Ethics and Research Committee of Shahid Beheshti University of Medical Science approved the study protocol.

**Fig 2:**
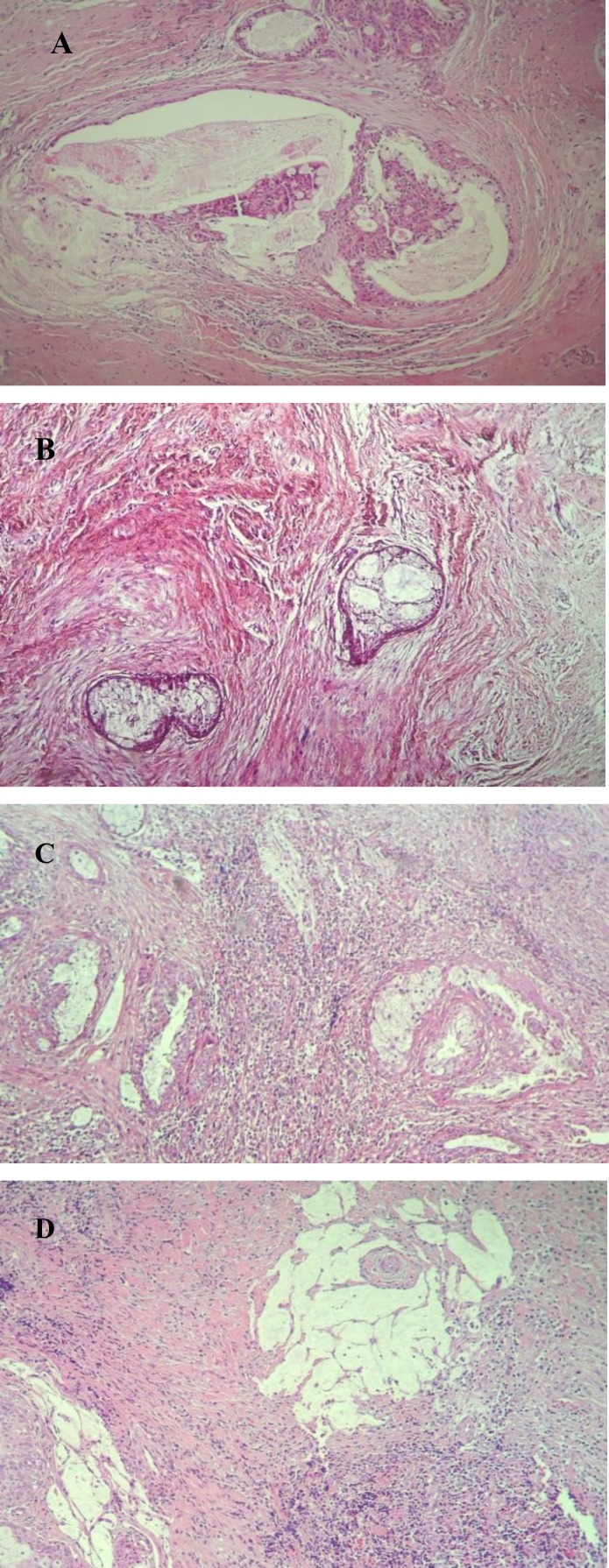
Low- grade Sclerosing mucoepidermoid carcinoma. Irregularly scattered cystic tumor islands in a dense sclerotic stroma composed of predominantly mucin-producing cells (A, B) and intermediate cells (C). Small pools of extravasated mucin and significant chronic inflammatory cell infiltrate are present (C, D

Microscopically, the vast majority of the tumor was composed of a densely sclerotic stroma, made up of haphazardly arranged collagen bands and fibroblasts, infiltrated by conspicuous chronic inflammatory cells especially at the peripheral zone with scattered irregular tumor islands located more centrally (Fig.2, A,B). 

Tumor nests were predominantly cystic and composed of a more prominent mucin-producing cells with vague intercellular bridges and intermediate cells with eosinophilic granular cytoplasms (Fig.2, A, C). Small pools and droplets of extra- vasated mucin were identified throughout the tumor (Fig. 2D). 

There was no nuclear anaplasia, perineural invasion, significant mitotic activity, or necrosis on serial sections. A focus of tumor invasion to peripheral skeletal muscle tissue was identified. The parotid gland showed partial parenchymal sclerosis, acinar atrophy, lymphoplasmacytic periductal and stromal infiltrates with scattered tumor islands and small mucin droplets extended to the margin of resection focally.

## Discussion

The behavior of mucoepidermoid carcinoma is strongly correlated with the clinical stage and histologic grade. Cure is possible, especially for low- and intermediate-grade tumors (2). Presented herein is another example of the rare sclerosing variant of mucoepidermoid carcinoma with low grade histology based on the presence of cysts (>80%), predominant mucinous cells, bland cytology, few mitotic figures, and absence of necrosis or perineurial invasion. Chan and Saw described the first case in 1987 and to our best knowledge only 23 additional cases of sclerosing MCEC with or without eosinophilia have since been described (3,4). There was one high grade case, four intermediate, and nineteen low grade cases including this present case (Table.1). 

As shown in the table, the reported cases (including this one) have 1- Generally occurred across a wide age range (17–79 years), 2- Shown a significant female preponderance (3:1), 3-Shown a predilection for the parotid gland (79%), 4- Been of intermediate sizes (average diameter, 2.5 cm). Although most cases have been low grade, there were cases of metastases (5).

As Fadare et al. noted (6), tumor size (more than 2cm) might be an important factor for predicting the prognosis of sclerosing MCEC. Metastases were found in both patient groups who received radiation therapy or not (5). In total, 11 cases have been reported previously that were associated with a dense infiltrate of eosinophils (3,8,10-13). Several conditions of the salivary glands may manifest morphologically with extensive stromal sclerosis or fibrosis, including sclerosing polycystic adenosis, hyalinizing clear cell carcinoma, mixed tumors, sclerosing sialadenitis, and polymorphous low-grade adenocarcinoma. Mucoepidermoid carci- nomas of the salivary glands also frequently show a sclerotic stroma (7). Of all the histological features observed in sclerosing MCEC, a central keloid-like sclerosis rimmed by peripheral lymphoid infiltration is unique enough to distinguish it from other sclerotic salivary lesions. However, since low grade sclerosing MCEC may be confused with benign lesions such as sclerosing sialadenitis especially on frozen section, pathologists must be aware of this rare variant of MCEC (5).

**Table 1:** Reported cases of sclerosing muciepidermoid carcinoma of the salivary gland.*

**Author & year**	**Age/Sex**	**Location**	**Size(cm)**	**Grade**	**Lymph**	**Eos**
Chan & Saw, 1987^4^	36/F	Parotid gland	2.2	Low	(+)	(-)
Muller & Barnes, 1997^8^	17/F	Parotid gland	2	intermediate	(+)	(+)
Muller & Barnes, 1997^8^	60/F	Parotid gland	2.5	intermediate	(+)	(+)
Sinha & Keogh, 1999^9^	65/M	Parapharyngeal space	5	High	(-)	(-)
Urano & Masato, 2002^10^	57/F	Parotid gland	2.5	Low	(+)	(+)
Urano & Masato, 2002^10^	43/F	Submandibular gland	2.5	Low	(+)	(+)
Fadare & Hileeto, 2004^6^	44/F	Parotid gland	4	Low	(-)	(-)
Ide & Obara, 2005^11^	28/M	Retromolar pad	2	Low	(+)	(+)
Heavner & Shah, 2006^12^	23/F	Parotid gland	2	Low	(+)	(+)
Veras & Sturgis, 2007^13^	70/F	Parotid gland	4	Low	(+)	(+)
Veras & Sturgis, 2007^13^	37/M	Parotid gland	2.2	Low	(+)	(+)
Veras & Sturgis, 2007^13^	49/F	Parotid gland	1.7	Low	(+)	(+)
Veras & Sturgis, 2007^13^	16/F	Parotid gland	2	intermediate	(+)	(+)
Aguiar & Bernardes, 2008^14^	43/F	Parotid gland	4	Low	(-)	(-)
Shinhar, 2010^15^	57/F	Parotid gland	2	intermediate	(+)	(-)
Mendelson & al-Macki, 2010^16^	21/F	Parotid gland	1.5	Low	(+)	(-)
Tian & Yakirevich, 2012^17^	42/F	Parotid gland	1.5	Low	(+)	_
Tian & Yakirevich, 2012^17^	52/F	Parotid gland	1.4	Low	(+)	_
Tian & Yakirevich, 2012^17^	62/F	Parotid gland	2	Low	(+)	_
Tian & Yakirevich, 2012^17^	28/M	Parotid gland	1.2	Low	(+)	_
Tian & Yakirevich, 2012^17^	65/F	Parotid gland	1.5	Low	(+)	_
Tian & Yakirevich, 2012^17^	32/M	Submandibular gland	1.1	Low	(+)	_
Takashi & Atsuji 2013^3^	79/M	Submandibular gland	5.6	Low	(+)	(+)
Present case	25/F	Parotid gland	2	Low	(+)	(-)

* Slightly modified from Takashi Tasaki et al.^3^

## Conclusion

In summary, we report another example of low grade sclerosing mucoepidermoid carcinoma with interesting presentation first as a posterior auricular mass - in contrast to usual parotid location - and believe it is necessary for pathologists to recognize a low grade sclerosing MCEC which has metastatic potential and is frequently misdiagnosed as a benign inflammatory lesion due to paucity of tumor islands.
